# Global responsibility vs. individual dreams: addressing ethical dilemmas created by the migration of healthcare practitioners

**DOI:** 10.1080/11287462.2020.1773054

**Published:** 2020-06-16

**Authors:** Fahmida Hossain

**Affiliations:** Center for Healthcare Ethics, Duquesne University, Pittsburgh, PA, USA

**Keywords:** Health practitioners, migration, global health, human rights, ethics

## Abstract

Background The migration of health care professionals from developing to developed countries is a trend. This migration benefits the destination countries but is quite often devastating to healthcare systems within the home countries. Skilled practitioners from developing countries forego opportunities in their homelands to migrate to developed countries. This leaves a vacuum of talent, weakening the health systems in the ‘home’ countries. Methods This piece analyzes the consequence of such migration through the lens of the four principles of Universal Declaration of Bioethics and Human rights (UDBHR): equality, justice and equity, solidarity and cooperation, and sharing of benefits. Results In the light of moral imagination and moral reflection, we can understand one another as global citizens. Policymakers must develop guides to restore balance and ensure equitable healthcare worldwide. Incorporating ethics education in medical schools and hospitals, implementing temporary migration visas, and helping home countries offer attractive compensation can address this concern. Conclusions Health is a universal human right; the well-being of all must be addressed without overly limiting the rights of practitioners to build the lives they imagine. On the other hand, practitioners should consider themselves global citizens and consider their ethical obligations when considering their migration.

## Background

Mobility is a hallmark of Globalization. Globalization – given the ease and opportunity for mobility – has made possible more expansive and sophisticated healthcare career options and opportunities worldwide. Health and medicine have become a globally shared platform, and health care practitioners now have expanded opportunities to move from one country to another. The majority of this migration is from developing countries to developed countries (Dodani & LaPorte, 2005). One of the byproducts of this movement is an ample supply of diverse practitioners in developed countries, which would otherwise run a shortage of skilled practitioners.

Furthermore, the intersection of different cultures improves the diversity mix within the workforce. These diverse workers bring with them cultural knowledge, which, in turn, enhances the efficacy and cultural sensitivity of the receiving system. Migrating practitioners also bring with them the best practices from their homelands, which are assimilated into the host healthcare systems. The practitioners who migrate are generally graduates of the finest schools in their home countries. The best, brightest, and most talented depart for foreign lands; this migration, in turn, dilutes the talent in their home countries. For example, the majority of all practitioners who migrated from Bangladesh to the USA, are graduates from Bangladeshi Medical Schools, and, in particular, from Dhaka Medical College, which is the best medical school in Bangladesh (Rahman & Khan, 2007). This one example is indicative of how the finest talent migrates abroad, brings best practices, and the desire to succeed to the receiving system. Most often, the benefit of the migration is one way: from the developing country to the developed country. Little of the new knowledge or knowledge from the established, receiving systems finds its way back to the developing countries. Thus, given current economic and social incentives for talented practitioner to leave their homelands, there are direct and negative consequences in terms of global health equality (Goštautaitė et al., 2018).

The voluntary migrating of practitioners is termed the “pull-up” approach. Pull-up migration is the quest to secure an improved personal lifestyle and economic well-being in a new country. This is also more broadly termed “consumption abroad.” This occurs when a patient, practitioner, or student from one country travels to another for superior treatment, an enhanced lifestyle, better education, or expanded professional opportunities (Parsi, 2008). These forms of migration create a talent vacuum, and, thus, the home country is without sufficient numbers of skilled practitioners. However, the migration of practitioners from developing countries to developed countries conveniently serves the growing demand for practitioners in developed countries (Davies, 2010). Also, the healthcare systems in developed countries not only gain practitioners who are “technically” talented and highly skilled; these incoming practitioners also add cultural and linguistic diversity to the receiving systems. Ninety-eight percent of all migrating practitioners are fluent in two or more languages (O'Reilly, 2019). Having such knowledgeable resources at hand is invaluable to help patients overcome what otherwise would be significant linguistic and cultural barriers to excellent care.

Even though the General Agreement on Trade and Services (GATS) permits or has no deterrents to the practice of consumption abroad, pull-up migration fosters global healthcare inequalities. In the course of the practitioners’ permanent migration to host countries, the home countries are deprived of their best practitioners – the lifeblood talent of their systems. Thus, the health systems in the home countries struggle to provide high quality, “developed world” healthcare due to the absence of a highly skilled workforce. Often this talent drain is so significant and severe that the “home” country cannot routinely satisfy necessary and basic levels of care (Davies, 2010). As of 2005, 11,000 licensed physicians were working in the USA (Rahman & Khan, 2007). Of those, 22.7% are international graduates, 27% have come from India. Also, high numbers of practitioners arrive from Nigeria, Syria, Pakistan, and Lebanon. This migration creates a deficiency of skilled practitioners in these poor “home” countries, all of which already have dangerously low doctor-patient ratios (O'Reilly, 2019).

According to the World Health Organization (WHO), the appropriate doctor-patient ratio should be 1:1000. The oil-rich country Qatar has the highest doctor-patient ratio, 77.4 practitioners per 1000 patients. Monaco (71.7) and Cuba (67.2) are the next two leaders (Loudermilk, 2018). However, in contrast, the doctor-patient ratio in Bangladesh is 5.26:10,000; there are only five doctors for every 10,000 patients (Alam, 2019). Nevertheless, even with such shortages, many high-talent Bangladeshi physicians migrate to developed countries for a better and more secure future.

There is reliable data – mainly out of Africa – which reveals that restrictions placed on free, outward practitioner migration improve the quality of healthcare services in the home countries (Mpofu et al., 2016). Also, almost all migrating practitioners are the country’s best and the brightest. This is due, in good part, to the strict and high standards which practitioners must meet in the receiving country. For example, in order to become a licensed physician in the United States, a practitioner must pass a detailed and highly rigorous comprehensive examination. Moreover, to be a licensed practitioner in the United States requires above-average English language proficiency (Rahman & Khan, 2007).

The migration of practitioners benefits the wealthy, privileged, and niche groups. The migration, on one level, preserves financial resources and infrastructure for the developed countries because they do not have to invest as much in educational services or training and development processes (Goštautaitė et al., 2018). The developed countries received a ready-made workforce in which the developing countries invested limited and precious resources. Thus, these migration benefits strengthen the rich and developed countries who already hold privileged positions in the healthcare hierarchy.

This disparity is even greater when considering sub-Saharan Africa. “Eleven percent of the world’s population live in sub-Saharan Africa and bear 24% of the global disease.” However, they only have 3% of the healthcare practitioners. In contrast, the United States, which has a significantly lower percentage of the global population and accounts for about 10% of all global illness, has 7% of all healthcare practitioners (Mpofu et al., 2016).

Migration directs resources away from the poor and underprivileged. From a pragmatic perceptive, this migration of healthcare practitioners retards the quest for universal, high-quality healthcare. It also has the consequence of overburdening national health systems. Thus, this practice makes the rich more powerful and destabilizes the public health infrastructure of poorer countries (Davies, 2010). The underprivileged continue to suffer due to the lack of readily available primary and essential treatment. Therefore, migration undermines public health systems and leaves the home countries struggling to provide consistent, readily available, quality care.

The wealthy actors in the healthcare system exploit the weaker players. This practice compromises the health of the poor in service to the broad and niche needs of the privileged. The developed countries do not “see” these inequities and seem baffled as to why the developing countries cannot provide world-class healthcare, oblivious to the fact that the best “local” talent has migrated to their countries (Have, 2016).

## Methods

This migration trend will be analyzed through the lens of the Principles within the Universal Declaration of Bioethics and Human rights (UDBHR). There are four Principles of the Universal Declaration of Bioethics and Human rights (UDBHR), which are applied to define the consequence of this migration as ethically and morally suspect. The principles are *equality*, *justice and equity, solidarity and cooperation,* and the *sharing of benefits*.

The Universal Declaration on Human Rights refers to all humans as one family (Article 1), and individuals have duties to the community (Article 29). Article 1 states that each individual has rights, but simultaneously all have an obligation to the community as “one human family.” Also, the notion of shared responsibility affirmed in the “United National Millennium Declaration” (2000), states there is a collective responsibility to maintain equality at a global level. Whereas the Principle of Human Rights focuses on the obligations collectively held to secure and ensure human rights, and to honor the belief that each person is entitled to receive the “shared responsibility” of the collective (Have, 2016). Regarding migration, health practitioners have the “right” to move freely in search of a preferred life. However, the right to emigrate must be qualified by and balanced with corresponding and corollary social obligations – especially regarding healthcare. The ethical and social obligations of healthcare professionals are rightfully held to a higher standard than many other professions – *primum non nocere* (Dwyer, 2007).

Healthcare professionals have collective ethical responsibilities to be global citizens without violating or suppressing the right to emigrate. Balancing such rights and obligations is a dilemma to be sure. Therefore, practitioners should first consider community obligations before deciding to migrate permanently.

According to the UDBHR *principle of solidarity and cooperation,* the migration of practitioners can be viewed as morally and ethically unjustified. The *principle of solidarity* dictates that all human beings act “as one family.” This necessarily requires each practitioner to view the self and others as global citizens. The *principle of solidarity* highlights the inalienable relationship between equals, which fosters cooperation and shared commonalities (Have, 2016). Such global solidarity founds the concept of a universal, moral community through which to critique the consequence of the migration of practitioners. According to this Principle, health is understood as a common good; thus, the health of all citizens – globally – are, by consequence, a common, shared concern. Healthcare equality is impossible if one’s actions cause others harm, no matter how seemingly benign. Therefore, practitioner migration must be seen as a direct violation of this Principle because it does not foster health as a global common good (eckenwiler et al., 2012).

According to the Principles of *equality*, and *justice and equity*, health benefits are human rights that should be available to all. The current and ever-increasing migration of practitioners creates an abundance of medical staff in developing countries. In contrast, the citizens in the home countries face a dearth of skilled practitioners (Have, 2016). This resultant inequity reveals that developed countries undermine social justice in developing countries and thus bring the issue of international justice and responsibility to the fore. The *maximum principle* and the principle of *equality of opportunity* address the fact that priority should be given to those most in need to meet the obligation that others live a healthy life. This ethical issue remains in the background concerning the effects of healthcare migration. Few institutions or practitioners take global responsibility for this fact (Dwyer, 2007). This injustice is experienced both at a global level and individual level. Those within the healthcare profession – and beyond – should agree on the concept of justice to ensure an equitable, peaceful, healthy world. As global citizens, healthcare practitioners (and administrators) are bound to respect the principle of Justice (Mpofu et al., 2016).

Coupled with the principle of *justice and responsibility* is the complementary principle of *sharing of benefits.* Benefit-sharing is founded on the notion that the benefits of service should be equally available to all. In the case of the migration of healthcare professionals, we see that the privileged increasingly benefit but are seemingly ever-less concerned about sharing these benefits with others (Have, 2016). Pull-up migration is morally problematic because the resources emerging countries invest in education do not bear local fruit because practitioners depart to practice elsewhere. An argument can be made that the nation that nurtures the physician holds limited claim to the practitioner’s skills and services. Likewise, citizens of the “home” countries overlook their obligations. To improve living standards for themselves and their families, they leave behind fallow fields. No one expects medical practitioners to be saints or be indentured servants, but each practitioner has a moral and ethical obligation to her country of origin (Parsi, 2008). Furthermore, developed countries are not compensating the home countries for the abundance of this *found* talent.

The UDBHR principles make clear how healthcare migration is morally and ethically wrong. This migration trend is responsible for an uneven, unequal global healthcare system. This bioethical issue requires, contends global health scholar Solomon Banatar, a reframing of our “moral imagination.” *Moral Imagination* addresses the critical thinking necessary for individuals to understand and embrace his or her obligation to society. Moral Imagination serves to sensitize an individual to the lives of all others, especially those less privileged or in crisis. Moral Imagination expands the circle of principled concerns and helps practitioners to recognize and respond to shared obligations to the human community (Have, 2016). Based on the concept of Moral Imagination, it is the duty of all to step forward and take responsibility for developing a better migration plan, which includes the subsequent actions to improve the lives of the underprivileged and those most affected by practitioner migration.

Also, according to John Rawls’ global justice theory, in case of inequalities, rather than redistribute assets to the poor, he calls for a process call “moral reflection.” Moral reflection appeals for common responsibilities, which extend to all, globally. Concerning the issue of health practitioner migration, Rawls contends that recruiting healthcare practitioners from developing countries is insensitive and a violation of human rights. From Rawls’ perspective, while practitioner migration may promote individual well-being, it discloses the collective responsibility practitioners and policymakers have to human welfare and the Earth’s well-being (Mpofu et al., 2016).

## Results

Migration is, of course, a human right, and any individual should be free to migrate in search of a better life. However, this right should also carry a corollary: obligation to the community. Pull-up migration reveals how healthcare is increasingly a service or commodity available to those who can afford it, which, in turn, offers economic benefits (profits) to the practitioners and the systems which benefit from the practitioners’ skills (Mpofu et al., 2016). However, ethically, healthcare should not be seen in an economic light. Health and wellbeing are human rights; the underprivileged are due equal access to healthcare. Given current and growing healthcare inequities, underprivileged populations should be given greater healthcare attention and resources – the purpose of healthcare is to heal. Therefore, in many ways, the extreme acceptance of pull-up migration is extraordinarily self-centered and ethically and morally dubious, because it ultimately benefits the privileged and causes increased hardships for those already underprivileged and struggling (Have, 2016).

Healthcare has become a form of global business. However, healthcare systems should remain distanced from the realm of “mere” business because the primary purpose of medicine is to heal – which goes ethically and morally beyond the monetary value of service. Even though on the surface migrations appears to be a national issue for the home country, it should be seen and understood in broader terms as a global ethical concern. Also, health *is* a universal human right, and the health and well-being of all should be addressed equally, everywhere, and for everyone – without compromising the rights of individuals or the responsibilities to the global community (Parsi, 2008).

Given the scope and force of globalization, we cannot directly restrict the migration of health professionals. However, it is possible to devise alternative methods to ensure that the home countries receive adequate healthcare and recompense for their initial investments in education and social infrastructure. It is not possible or wise to completely stop the “brain drain” of talent, but it is possible to replace the loss by “Brain Circulation”. Brain Circulation is an innovative approach that the Chinese government has taken in order to entice the lost talent to come home, even for a short or scheduled period of time (Nair & Webster, 2013).

## Recommendations

Establishing international agreements for benefit-sharing would promote the ethical migration of health practitioners. Such benefit-sharing could take monetary and non-monetary forms. Monetary benefit-sharing should entail that receiving organizations in the developed and privileged countries supply financial assets to the developing countries to support healthcare and to ensure sufficient numbers of medical practitioners are trained for their country’s needs. A joint research fund, access fees, or any other modes of monetary assistance could be given to the home country to reduce healthcare inequality (Have, 2016).

Nonmonetary benefits (teaching and training) could also be offered to the home country as a way of support and compensation for accepting migrated practitioners. Healthcare practitioners and organizations, furthermore, should share textbooks, professional journals, and other educational materials as a means of support and ensure the most up-to-date references are at hand. Holding seminars, workshops, and training in the home countries is another way to mitigate the effects of the talent drain; this would be an effective means to develop home country practitioners without the need for students to travel abroad. Implementing telemedicine projects could also be a highly effective means of training and skill-building (Rahman & Khan, 2007). By applying web-based technologies, it is possible to schedule sophisticated consultations almost anywhere on the planet. Such consultations would provide at-need expertise not available in the home country. China set an example by becoming the first developing country to participate in the Human Genome project as a way to collaborate with the developed countries (Dodani & LaPorte, 2005). Targeted educational opportunities like this are practical and useful ways to mitigate or counteract the effects of brain-drain.

A Healthcare Ethics Curriculum also should be implemented in all in medical schools. The incorporation of ethical education that highlights the global responsibilities of practitioners must be included in medical curriculums internationally. Embedding the belief in global citizenship within the teaching curriculum will help practitioners embrace their social and global obligations when making “personal” choices. Whether providing benefits or sharing best practices, policymakers must be keenly aware of the social and cultural context of a particular country. Ethics-oriented training and workshops should be organized by developing countries to manage or moderated the migration process (Yuksekdag, 2017).

To mitigate the negative consequence of pull-up migration, policies and practices should be established to offer temporary or limited migration opportunities to practitioners (Parsi, 2008). One option is to provide a short-term visa that would require medical practitioners, after a specific period, to return to their home countries to provide health care services – much in line with the notion of country service. Another way might be specific periods of “service time” required by the home country following the completion of medical education and training. Such practices would ensure that medical tourism and pull-up migration do not solely benefit the privileged but also help local systems (Davies, 2010).

A practice of note is that the international agreements between the Organization for Economic Cooperation and Development (OECD) countries. Through this agreement, health practitioners must fulfill specific and prescribed requirements in order to migrate to another country. Doing so will ensure the home country continues to reap benefit from its investment in training physicians (Forcier et al., 2004). Of course, it is understood that if these policies and rules become too restrictive, they could interfere with the human right of self-determination, but it is possible to frame this debate by addressing the responsibilities established by the human rights principles that highlight the universal obligation to the other.

However, one of the soundest approaches home countries can take is to build infrastructure attractive enough to ensure local gems stay “home.” Reverse brain-drain programs should be implemented that offer attractive financial packages and satisfactory, high-quality work environments to lure or keep talent “home” (Mpofu et al., 2016). Brain Circulation programs should also become a great source of the triangular flow of knowledge and expertise (Nair & Webster, 2013).

As a means to retain skilled talent, effective non-financial reward systems should be developed, which ensures that practitioners work in dignified and motivating settings (Goštautaitė et al., 2018). However, this is problematic for developing or emerging countries because they have limited resources to construct attractive strategies and facilities to retain skilled professionals. In these instances, others – receiving-country systems and success migrated practitioners – must embrace the role of global citizenship and “payback” to the home countries. Developed countries that benefit from migration should likewise also pay back to the countries of origin in associated forms of political or trade support. When this occurs, countries can route these new resources to develop policies that serve to retain their most skilled workers. Thailand and Ireland are good examples of countries that have successfully found policy ways to avoid migratory brain drain of medical talent (Parsi, 2008).

## Conclusion

The debate on the migration of health practitioners is a dominant issue for global bioethics. Few would argue to deny the right of an individual to seek a better standard of living. However, this right does not free those leaving their homelands from the moral and ethical obligation “to the other” – especially to those negatively affected by the practitioner’s decision to migrate. Furthermore, healthcare practitioners are obligated to adhere to the highest, guiding ethical and moral standards. Migration has direct and profound ethical and moral ramifications for the migrant, the receiving country, and the country left behind. Fair and socially based policies must be developed that counter the radical argument that individuals have an unfettered right to live her life as she sees fit. Practitioners and the policymakers must consider themselves global citizens and embrace and respond to the stated and agreed upon moral and ethical obligations required to the equity of global healthcare. To construct, implement, and safeguard equitable healthcare, practitioners and policymakers – at a global scale – must maintain a firm balanced approach between individual rights and social obligations.

Practitioners have the right to build lives that meet life and career goals, wherever this may be; however, this freedom comes with an important caveat: as human beings, we coexist on *this* Earth – global citizens, who have moral and ethical obligations to the other.
